# Serum and Salivary IgG and IgA Response After COVID-19 Messenger RNA Vaccination

**DOI:** 10.1001/jamanetworkopen.2024.8051

**Published:** 2024-04-23

**Authors:** Guy Gorochov, Jacques Ropers, Odile Launay, Karim Dorgham, Omaira da Mata-Jardin, Said Lebbah, Christine Durier, Rebecca Bauer, Anne Radenne, Corinne Desaint, Louis-Victorien Vieillard, Claire Rekacewicz, Marie Lachatre, Béatrice Parfait, Frédéric Batteux, Philippe Hupé, Läétitia Ninove, Maeva Lefebvre, Anne Conrad, Bertrand Dussol, Zoha Maakaroun-Vermesse, Giovanna Melica, Jean-François Nicolas, Renaud Verdon, Jean-Jacques Kiladjian, Paul Loubet, Catherine Schmidt-Mutter, Christian Dualé, Séverine Ansart, Elisabeth Botelho-Nevers, Jean-Daniel Lelièvre, Xavier de Lamballerie, Marie-Paule Kieny, Eric Tartour, Stéphane Paul

**Affiliations:** 1 Sorbonne Université, Institut National de la Santé et de la Recherche Médicale (INSERM), Centre d’Immunologie et des Maladies Infectieuses (CIMI), Département d’Immunologie, Assistance Publique–Hôpitaux de Paris (AP-HP), Hôpital Pitié-Salpêtrière, Paris, France; 2 INSERM, Institut Pierre Louis d’Epidémiologie et de Santé Publique, AP-HP, Hôpital Pitié-Salpêtrière, Département de Santé Publique, Unité de Recherche Clinique Paris Sciences et Lettres (PSL)–CFX, Sorbonne Université, Paris, France; 3 Université Paris Cité, INSERM, Centre d’Investigation Clinique (CIC) 1417 Cochin Pasteur, French Clinical Research Infrastructure Network, Innovative Clinical Research Network in Vaccinology, APHP, Hôpital Cochin, Paris, France; 4 INSERM SC10-US019, Villejuif, France; 5 AP-HP, Hôpitaux Universitaires Pitié-Salpêtrière–Charles Foix, Unité de Recherche Clinique des Hôpitaux Universitaires Pitié-Salpêtrière, Paris, France; 6 AP-HP, Hôpital Cochin, Fédération des Centres de Ressources Biologiques–Plateforme de Ressources Biologiques Centre de Ressources Biologique Cochin, Paris, France; 7 AP-HP, Hôpital Cochin, Service d’Immunologie Biologique et Plateforme d’Immunomonitoring Vaccinal, Paris, France; 8 Institut Curie, PSL Research University, INSERM U900, MINES ParisTech, PSL, Paris, France; 9 Centre National de la Recherche Scientiﬁque (CNRS), Unité Mixte de Recherche (UMR) 144, Paris, France; 10 Research Institute for Sustainable Development 190, INSERM 1207, Institut Hospitalier Universitaire Méditerranée Infection, Unité des Virus Émergents, Aix Marseille Université, Marseille, France; 11 Centre Hospitalier Universitaire (CHU) de Nantes, INSERM CIC 1413, Maladies Infectieuses et Tropicales, Centre de Prévention des Maladies Infectieuses et Transmissibles, Nantes, France; 12 Département des Maladies Infectieuses et Tropicales, Hôpital de la Croix-Rousse, Hospices Civils de Lyon, Centre International de Recherche en Infectiologie (CIRI), INSERM U1111, Université Claude Bernard Lyon I, CNRS, UMR5308, École Normale Supérieure de Lyon, Université Lyon, Lyon, France; 13 CIC 1409, INSERM–Hôpitaux Universitaires de Marseille–Aix Marseille Université, Hôpital de la Conception, Marseille, France; 14 Centre de Vaccination CHU de Tours, CIC 1415, INSERM, Centre Hospitalier Régional et Universitaire de Tours, Tours, France; 15 Service d’Immunologie Clinique et Maladies Infectieuses, AP-HP, Hôpital Henri Mondor, Créteil, Centre d’Investigation Clinique 1430 INSERM, AP-HP, Hôpital Henri Mondor, Créteil, France; 16 CIRI, INSERM U1111, Université Claude Bernard Lyon I, Lyon, CHU Lyon-Sud, Pierre-Bénite, France; 17 Service de Maladies Infectieuses, CHU de Caen, Dynamicure INSERM UMR 1311, Normandie Université, University of Caen Normandy, Caen, France; 18 Université Paris Cité, AP-HP, Hôpital Saint-Louis, Centre d’Investigations Cliniques, INSERM, CIC 1427, Paris, France; 19 Virulence Bactérienne et Maladies Infectieuses, INSERM U1047, Department of Infectious and Tropical Diseases, CHU 37 Nîmes, Université de Montpellier, Nîmes, France; 20 INSERM CIC 1434, CHU Strasbourg, Strasbourg, France; 21 CIC, INSERM CIC1405, CHU Clermont-Ferrand, Clermont-Ferrand, France; 22 CIC 1412, INSERM, CHU Brest, Brest, France; 23 INSERM CIC 1408, Axe Vaccinologie, CHU de Saint-Étienne, Service d’Infectiologie, Saint-Étienne, France; 24 INSERM U955, Vaccine Research Institute, Créteil, France; 25 INSERM 101, Paris, France; 26 AP-HP, Hôpital Européen Georges Pompidou, INSERM U970, Paris Cardiovascular Research Center, Université Paris Cité, Paris, France; 27 INSERM, U1111, CNRS, UMR 5308, CIRI-GIMAP, Université Claude Bernard Lyon 1, Université Jean Monnet, Immunology and Immunomonitoring Laboratory, iBiothera, CIC 1408, Saint-Étienne, France

## Abstract

**Importance:**

There is still considerable controversy in the literature regarding the capacity of intramuscular messenger RNA (mRNA) vaccination to induce a mucosal immune response.

**Objective:**

To compare serum and salivary IgG and IgA levels among mRNA-vaccinated individuals with or without previous SARS-CoV-2 infection.

**Design, Setting, and Participants:**

In this cohort study, SARS-CoV-2–naive participants and those with previous infection were consecutively included in the CoviCompare P and CoviCompare M mRNA vaccination trials and followed up to day 180 after vaccination with either the BNT162b2 (Pfizer-BioNTech) vaccine or the mRNA-1273 (Moderna) vaccine at the beginning of the COVID-19 vaccination campaign (from February 19 to June 8, 2021) in France. Data were analyzed from October 25, 2022, to July 13, 2023.

**Main Outcomes and Measures:**

An ultrasensitive digital enzyme-linked immunosorbent assay was used for the comparison of SARS-CoV-2 spike-specific serum and salivary IgG and IgA levels. Spike-specific secretory IgA level was also quantified at selected times.

**Results:**

A total of 427 individuals were included in 3 groups: participants with SARS-CoV-2 prior to vaccination who received 1 single dose of BNT162b2 (Pfizer-BioNTech) (n = 120) and SARS-CoV-2–naive individuals who received 2 doses of mRNA-1273 (Moderna) (n = 172) or 2 doses of BNT162b2 (Pfizer-BioNTech) (n = 135). The median age was 68 (IQR, 39-75) years, and 228 (53.4%) were men. SARS-CoV-2 spike-specific IgG saliva levels increased after 1 or 2 vaccine injections in individuals with previous infection and SARS-CoV-2–naive individuals. After vaccination, SARS-CoV-2–specific saliva IgA levels, normalized with respect to total IgA levels, were significantly higher in participants with previous infection, as compared with the most responsive mRNA-1273 (Moderna) recipients (median normalized levels, 155 × 10^−5^ vs 37 × 10^−5^ at day 29; 107 × 10^−5^ vs 54 × 10^−5^ at day 57; and 104 × 10^−5^ vs 70 × 10^−5^ at day 180 [*P* < .001]). In contrast, compared with day 1, spike-specific IgA levels in the BNT162b2-vaccinated SARS-CoV-2–naive group increased only at day 57 (36 × 10^−5^ vs 49 × 10^−5^ [*P* = .01]). Bona fide multimeric secretory IgA levels were significantly higher in individuals with previous infection compared with SARS-CoV-2–naive individuals after 2 antigenic stimulations (median optical density, 0.36 [IQR, 0.16-0.63] vs 0.16 [IQR, 0.10-0.22]; *P* < .001).

**Conclusions and Relevance:**

The findings of this cohort study suggest that mRNA vaccination was associated with mucosal immunity in individuals without prior SARS-CoV-2 infection, but at much lower levels than in previously infected individuals. Further studies are needed to determine the association between specific saliva IgA levels and prevention of infection or transmission.

## Key Points

**Question:**

Can intramuscularly administered messenger RNA vaccines induce mucosal immunity?

**Findings:**

In this cohort study of 427 vaccinated individuals, a modest increase in spike-specific IgA levels in saliva of SARS-CoV-2–naive individuals having received the Moderna or Pfizer-BioNTech messenger RNA vaccines was observed using ultrasensitive digital enzyme-linked immunosorbent assay. This increase persisted after Moderna vaccination, and previously infected individuals demonstrated a stronger mucosal IgA response to vaccination.

**Meaning:**

Specific salivary IgA can be detected after messenger RNA vaccination solely, but to much lower levels than in previously infected individuals.

## Teaser

This cohort study compares individuals with previous SARS-CoV-2 infection and SARS-CoV-2–naive individuals for their saliva and serum responses after infection and vaccination or vaccination alone.

## Introduction

IgA is monomeric in human serum but is also produced locally in mucosal tissues mostly under dimeric or even polymeric forms and released associated with the secretory component as secretory IgA (SIgA).^1^ Monomeric IgA is also present in saliva following passive transport from blood.^1^ Saliva represents an easily accessible body fluid permitting probing of IgA mucosal immunity. Sterlin et al^2^ previously showed that potent SARS-CoV-2–neutralizing IgA antibodies are rapidly produced following infection, even before IgG antibodies, although their levels decline and fail to be detected in saliva around 6 months after symptom onset. Recent studies^3,4^ suggest that SARS-CoV-2 spike-specific mucosal IgA antibodies could provide protection against breakthrough infection. However, it remains unclear whether messenger RNA (mRNA)–based vaccines can induce such SIgA responses. Several studies conclude that humoral mucosal immunity is largely driven by previous infection, with little impact of vaccination,^4,5,6^ while others suggest that mRNA vaccination can by itself elicit long-lasting anti–SARS-CoV-2 mucosal IgA responses.^7,8,9,10,11^ Herein, we compared individuals with previous SARS-CoV-2 infection and SARS-CoV-2–naive individuals included in the CoviCompare P and CoviCompare M vaccination trials^12^ for their saliva and serum responses after 2 different modes of antigenic challenge: infection and vaccination or vaccination alone.

## Methods

The CoviCompare P and CoviCompare M trials received ethical approval from Comité De Protection des Personnes Ile De France 1. All participants provided written informed consent. The study followed the Strengthening the Reporting of Observational Studies in Epidemiology (STROBE) reporting guideline.

### Participants

From February 19 to June 8, 2021, participants without prior SARS-CoV-2 infection received either 2 doses of the mRNA-1273 (Moderna) vaccine in the CoviCompare M trial or the BNT162b2 (Pfizer-BioNTech) vaccine in the CoviCompare P trial at 28 days apart, whereas participants with a documented prior SARS-CoV-2 infection and positive serologic findings received only 1 dose of BNT162b2 (Pfizer-BioNTech) in accordance with the French guidelines at the time. In each trial, participants were healthy adults in stable medical condition. Samples were collected at multiple times: before the first vaccine administration (day 1), before the second vaccine administration (day 29), 28 days after the second vaccine dose (day 57), and 6 months after the first dose (day 180). Saliva was collected in a cup and serum was collected by venipuncture. All biospecimen were stored at −80°C prior to analysis.

Overall, 180 and 267 patients were included in the CoviCompare M and P trials, respectively. For the purpose of mucosal antibody analyses, 20 patients were excluded because (1) the second vaccine dose was delayed by more than 14 days after the planned date (n = 2); (2) anti–nucleocapsid levels were either missing before day 180 or were above the positivity threshold (n = 13); or (3) a SARS-CoV-2 infection was documented (using polymerase chain reaction analysis) at any time until day 180 (n = 5).

After these exclusions, 427 participants were included in these analyses, among whom 120 had a documented SARS-CoV-2 infection at least 5 months prior to their single dose of BNT162b2 vaccine (Pfizer-BioNTech) at the beginning of the pandemic, therefore at a period when only ancestral wild-type SARS-CoV-2 (D614G) was circulating in France (Table). The remaining 307 patients had no prior SARS-CoV-2 infection and received either 2 doses of mRNA-1273 (Moderna) (n = 172) or 2 doses of BNT162b2 (Pfizer-BioNTech) (n = 135).

**Table.** Participant Characteristics

Characteristic	Participant group				*P* value^a^
	All (n = 427)	Infection naive with mRNA-1273 (Moderna) (n = 172)	Infection naive with BNT162b2 (Pfizer-BioNTech) (n = 135)	Previous infection BNT162b2 (Pfizer-BioNTech) (n = 120)	
Age, median (IQR), y	68 (39-75)	69 (40-75)	68 (39-75)	67 (37-70)	.05
Age group, No. (%)					
18-45	152 (36)	55 (32)	47 (35)	50 (42)	.20
65-74	168 (39)	69 (40)	50 (37)	49 (41)	
>74	107 (25)	48 (28)	38 (28)	21 (18)	
Sex, No. (%)					
Men	228 (53)	98 (57)	68 (50)	62 (52)	.49
Women	199 (47)	74 (43)	67 (50)	58 (48)	
Body mass index, median (IQR)^b^	25 (22-27)	25 (22-27)	24 (22-27)	25 (22-29)	.11
First dose injection date, range	February 19 to June 8, 2021	February 19 to April 16, 2021	March 10 to May 4, 2021	March 15 to June 8, 2021	NA

Abbreviation: NA, not applicable.

^a^ Calculated using Kruskal-Wallis rank sum test or Fisher exact test.

^b^ Calculated as weight in kilograms divided by height in meters squared.

### Quantification of Total Saliva IgA and IgG by Enzyme-Linked Immunosorbent Assay

For quantification of total IgA levels, high-binding 96–half-well plates (2310M; Nunc) were coated overnight at 4°C with 4 μg/mL of polyclonal goat anti–human IgA (I1261; Sigma-Aldrich) in phosphate-buffered solution (PBS). Serial dilutions of saliva in 5% skim milk in PBS, supplemented with 0.1% polysorbate 20 (Tween 20; Sigma-Aldrich), were added and plates were subsequently incubated for 1.5 hours at room temperature. Plates were then washed and incubated for 1 hour at room temperature with horseradish peroxidase (HRP)–conjugated polyclonal goat anti–human IgA (A0295; Sigma-Aldrich) diluted at 1:10 000 in PBS 5% milk. Bound antibodies were detected using tetramethylbenzidine substrate (T0440; Sigma-Aldrich). The color reaction was stopped with 0.5M sulfuric acid (H_2_SO_4_) after a 10-minute incubation, and the absorbance was measured at 450 nm in an enzyme-linked immunosorbent assay (ELISA) plate reader (Varioskan; Thermo Fisher Scientific Inc). Serial dilutions of prequantified saliva pool and monoclonal IgA were used for the generation of standard curves and measurement of concentration.

For measurement of total IgG, goat antihuman IgG Fc fragment antibody (A80-104A; Bethyl Laboratories) was coated overnight at 4°C (100 μL per well at 1 μg/mL in PBS) in 96-well plates (Immuno Maxisorp plates; Nunc). Plates were blocked for 1 hour in combined PBS and 3% bovine serum albumin, and a 500-fold dilution of saliva in combined PBS–3% bovine serum albumin was added and incubated for 1 hour at room temperature. Plates were washed 5 times with PBS–0.5% polysorbate 20 (Sigma-Aldrich) and incubated for 1 hour at room temperature with goat anti–human IgG Fc fragment HRP conjugated (A80-104P; Bethyl Laboratories) diluted to 1:150 000. After washing, 100 μL of tetramethylbenzidine (002023; Life Technologies) was added to each well, the reaction was stopped with 50 μL of 0.5M H_2_SO_4_ after 10 minutes of incubation, and the absorbance was measured at 450 nm in a microplate reader. Calculation of individual IgG concentrations was based on standard curves prepared with a commercial standard (N Protein Standard SL, model OQIM13; Siemens Healthcare Diagnostics AG).

### SARS-CoV-2 Spike- and Nucleocapsid-Specific Serum Antibody Quantification by ELISA

Serum samples were tested for anti–SARS-CoV-2 IgG and IgA antibodies directed against the S1 domain of the spike protein of the virus using a commercial ELISA kit (EUROIMMUN). The kit is based on the detection of the Wuhan spike protein. Quantitative results were expressed in standardized units (binding antibody units [BAU] per milliliter). Serum IgG levels against SARS-CoV-2 nucleocapsid protein were detected using the V-PLEX COVID-19 serology kit (Meso Scale Discovery) and interpreted according to the manufacturer’s instructions.

### SARS-CoV-2 Spike-Specific Saliva Antibody Quantification by Digital ELISA

SARS-CoV-2 spike IgG antibody levels were measured as previously described^13,14^ by using a SARS-CoV-2 spike IgG assay (Simoa Advantage kit; Quanterix Corporation) on the SR-X Platform (Quanterix Corporation) according to the manufacturer’s instructions. Saliva samples were diluted 100-fold in sample diluent provided in the kit. We used an ultrasensitive digital ELISA for the measurement of specific anti–SARS-CoV-2 spike IgA mucosal responses, which allowed a better discrimination from background signals than classical ELISA performed in parallel on the same samples. The SARS-CoV-2 spike IgA antibody assay was developed using reagents provided in the SARS-CoV-2 spike IgG assay kit (Quanterix Corporation). Saliva samples were diluted 100-fold in sample diluent plus 0.5% polysorbate 20. Biotinylated antihuman IgA (clone REA1014; Miltenyi Biotec) diluted in sample diluent at 100 ng/mL was used as detector reagent. Streptavidin-β-galactosidase (SβG) was diluted to 25 pM in SβG diluent (SR-X 103158; Quanterix Corporation). Calibrator reagent consisted of recombinant anti–SARS-CoV-2 spike IgA (clone CR3022; Diaclone) diluted in sample diluent in a 4-fold dilution series (range, 0.05-200 ng/mL). Signals are expressed in BAU per milliliter. The lower limit of quantification was estimated to be 10 BAU/mL (eMethods in Supplement 1).

To avoid biases related to the fact that total IgA concentration within saliva is variable in samples from the same individual collected at different times, depending on various factors such as mode of sample collection and stress,^1^ we normalized SARS-CoV-2 spike-specific IgA and IgG titers to the total IgA and IgG concentrations, respectively, within each saliva sample. Normalization of SARS-CoV-2 spike-specific saliva antibodies was calculated as follows: Normalized IgG = Anti–spike IgG/Total IgG and Normalized IgA = Anti–spike IgA/Total IgA.

### Secretory SARS-CoV-2 Specific IgA Quantification by ELISA

To compare the best response in saliva samples among SARS-CoV-2–naive participants (ie, the group receiving mRNA-1273 vaccine) with individuals who had a previous infection, for secretory piece-bound SIgA only, we selected groups exposed to 2 antigenic stimulations: previous infection and SARS-CoV-2–naive individuals after a single or a second injection, respectively. High-binding 96–half-well plates (2310M) were coated overnight at 4°C with 1 μg/mL of S1 spike protein (Wuhan strain) in carbonate coating buffer. Saturation of the coated plates were performed with 5% skim milk in PBS during 90 minutes at 37°C. Serial dilutions of saliva in 5% skim milk in PBS supplemented with 0.1% polysorbate 20 were added, and plates were subsequently incubated overnight at 4°C. Plates were then washed and incubated for 1 hour at room temperature with mouse antihuman secretory piece (1:1000 dilution; MilliporeSigma). After washing, plates were then revealed with HRP-conjugated polyclonal goat antimouse IgG (Invitrogen). Bound antibodies were detected using tetramethylbenzidine substrate (T0440; Sigma-Aldrich). The color reaction was stopped with 0.5M H_2_SO_4_ after 10-minute incubation, and the absorbance was measured at 450 nm in an ELISA plate reader (Varioskan).

### Statistical Analysis

Data were analyzed from October 25, 2022, to July 13, 2023. Data were expressed as medians (IQRs) for continuous data and as frequencies and proportions for categorical data. For specific IgG and IgA levels, we computed a cutoff arbitrarily set at the mean plus 1 SD of measurements performed at baseline in SARS-CoV-2–naive participants. Comparisons between groups were performed using the Wilcoxon rank sum test (with stratification on age as sensitivity analysis) and Fisher exact test for categorical data. Within groups, comparisons between different times were performed using Wilcoxon rank sum test for paired data or McNemar test for proportions of participants above threshold. Fold changes of the geometric means were estimated with their 95% CI. All tests were performed on log_10_-transformed data at the 2-sided 5% significance level (*P* < .05). Correlations between salivary and serum antibody levels were estimated using the Spearman correlation coefficient. All calculations were performed using R software, version 4.1.1 (R Program for Statistical Computing).

## Results

We included 427 participants at the beginning of the pandemic, a period when only ancestral wild-type SARS-CoV-2 (D614G) was circulating in France. Of these, 120 participants had a documented SARS-CoV-2 infection prior to their single dose of BNT162b2 vaccine. The median age was 68 (IQR, 39-75) years; 228 participants (53.4%) were men and 199 (46.6%) were women. Race and ethnicity data were not collected, in conformity to French law.

**Figure 1.**
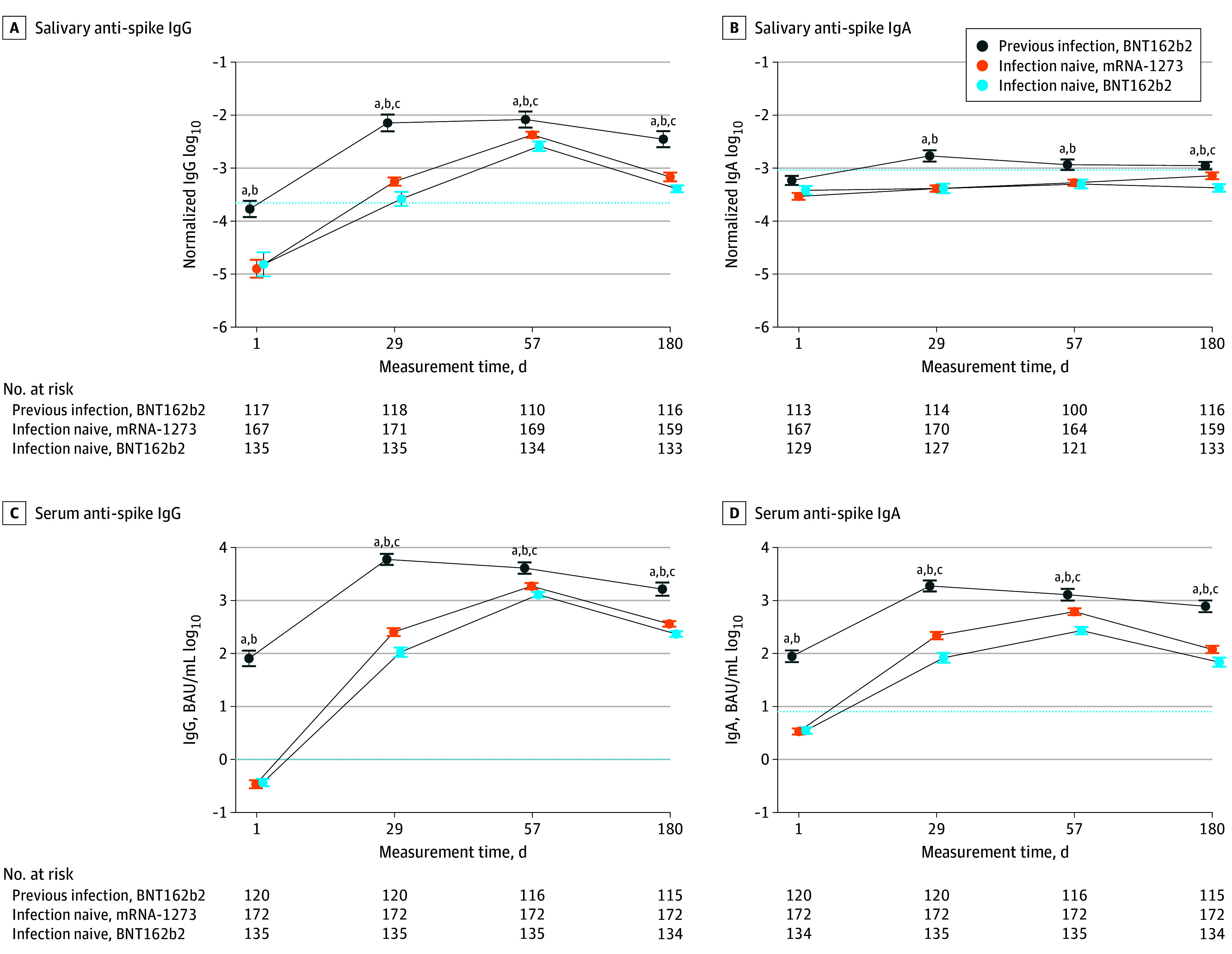
Longitudinal Evolution of Serum and Salivary Anti–Spike IgG and IgA Following Messenger RNA (mRNA) Vaccination in Individuals With or Without Previous Exposure to SARS-CoV-2 Evolution over time in individuals with previous SARS-CoV-2 infection receiving the BNT162b2 (Pfizer-BioNTech) vaccine, SARS-CoV-2–naive individuals receiving the mRNA-1273 (Moderna) vaccine, and SARS-CoV-2–naive individuals receiving BNT162b2 (Pfizer-BioNTech) of median log_10_ levels of salivary and serum anti–spike IgG or IgA at days 1, 29, 57, and 180 after vaccination. Error bars indicate 95% CI. Dotted lines indicate threshold values computed as mean plus 1 SD of measurements made at day 1 among patients without prior infection. Results of comparison between groups at each time (Wilcoxon rank sum test) are shown. ^a^*P* ≤ .001, previous infection vs infection naive with mRNA-1273 vaccine. ^b^*P* ≤ .001, previous infection vs infection naive with BNT162b2 vaccine. ^c^*P* ≤ .001, infection naive with mRNA-1273 vaccine vs infection naive with BNT162b2 vaccine.

Serum baseline SARS-CoV-2 spike-specific IgG and IgA levels (Figure 1 and eTable 1 in Supplement 1) were elevated in individuals with previous infection and increased to higher levels following vaccination, compared with SARS-CoV-2–naive individuals. Among SARS-CoV-2–naive patients, at all times following vaccination, serum anti–spike IgG and IgA levels were significantly higher in the mRNA-1273 compared with the BNT162b2 groups (IgG: 285.4 vs 117.5 BAU/mL at day 29, 2090.9 vs 1292.4 BAU/mL at day 57, and 395.4 vs 247.9 BAU/mL at day 180 [*P* < .001 for all comparisons]; IgA: 217.1 vs 74.4 BAU/mL at day 29, 603.9 vs 249.4 BAU/mL at day 57, and 119.2 vs 63.0 BAU/mL at day 180 [*P* < .001 for all comparisons]) (Figure 1).

**Figure 2.**
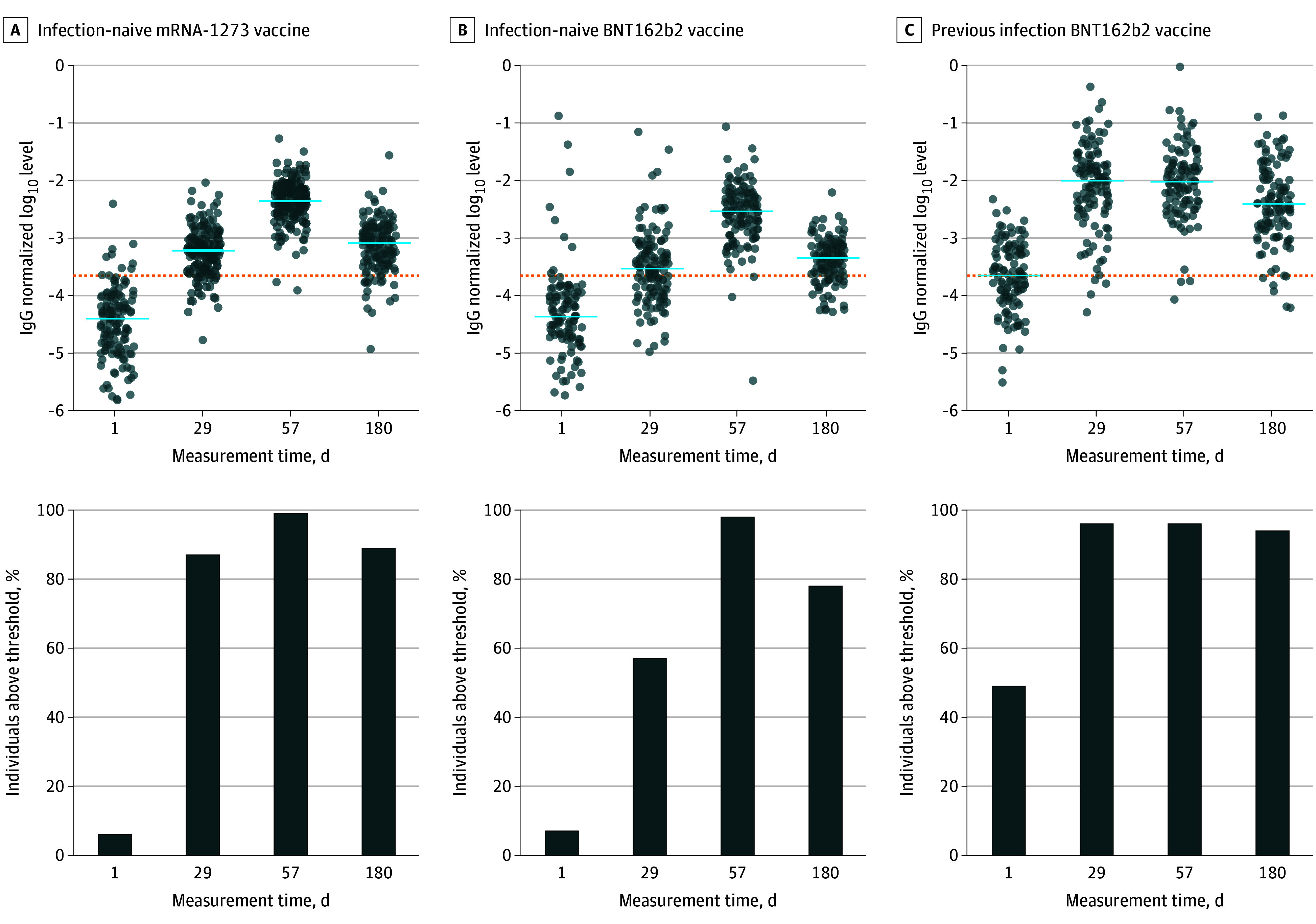
Salivary Anti–Spike IgG Response Following Messenger RNA (mRNA) Vaccination Evolution over time in SARS-CoV-2–naive individuals receiving the mRNA-1273 (Moderna) vaccine or BNT162b2 (Pfizer-BioNTech) vaccine and previously infected individuals receiving BNT162b2 (Pfizer-BioNTech) of median log_10_ salivary anti–spike IgG normalized on total salivary IgG levels at days 1, 29, 57, and 180 after vaccination, measured using digital enzyme-linked immunosorbent assay. Dotted lines indicate threshold values computed as mean plus 1 SD of measurements made at day 1 among patients without prior infection. Horizontal blue bars indicate median values. Results of the comparison between time points (Wilcoxon rank sum test) are provided in eTable 5 in Supplement 1.

A significant increase in specific IgG saliva levels was mostly detected after vaccination in previously infected individuals, but also in SARS-CoV-2–naive individuals after each injection (Figure 2). Levels of salivary and serum anti–spike IgG antibodies were correlated in all 3 groups and at all times measured (eFigure 1 in Supplement 1), consistently with the known predominant systemic origin of saliva IgG.^1^

**Figure 3.**
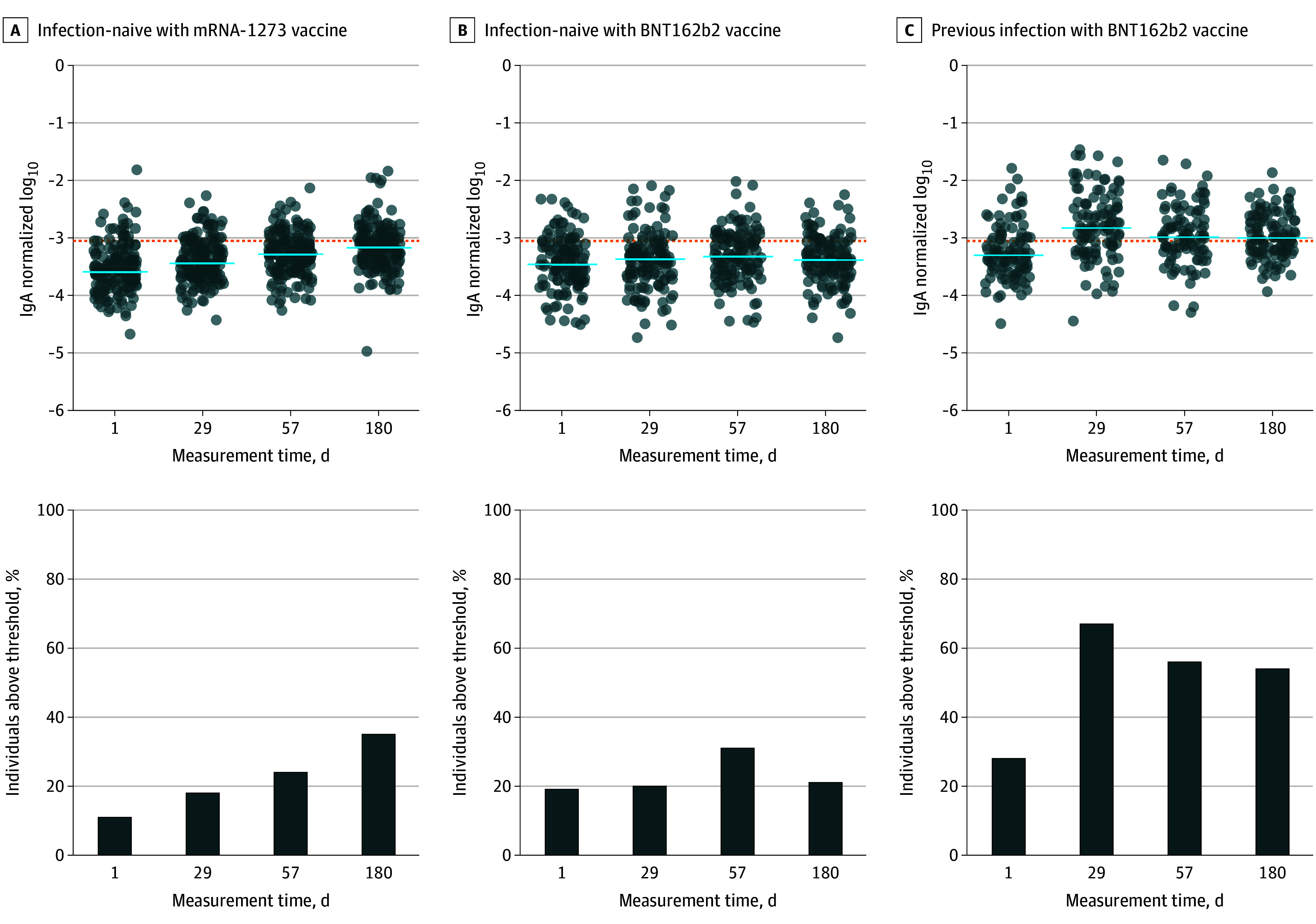
Salivary Anti–Spike IgA Response Following Messenger RNA (mRNA) Vaccination Evolution over time in SARS-CoV-2–naive individuals receiving the mRNA-1273 (Moderna) vaccine or the BNT162b2 (Pfizer-BioNTech) vaccine and previously infected individuals receiving BNT162b2 (Pfizer-BioNTech) of median log_10_ salivary anti–spike IgA levels normalized on total salivary IgA at days 1, 29, 57, and 180 after vaccination, measured using digital enzyme-linked immunosorbent assay (taking into account both monomeric and multimeric secretory IgA). Dotted lines indicate threshold values computed as mean plus 1 SD of measurements made at day 1 among patients without prior infection. Horizontal blue bars indicate median values. Results of the comparison between time points (Wilcoxon rank sum test) are provided in eTable 5 in Supplement 1.

The highest levels of SARS-CoV-2 spike-specific saliva IgA were detected in patients with previous infection 1 month after vaccination and slightly declined afterward (medians of normalized levels: 155 × 10^−5^ [IQR, 69 × 10^−5^ to 387 × 10^−5^] at day 29; 107 × 10^−5^ [IQR, 63 × 10^−5^ to 217 × 10^−5^] at day 57; and 104 × 10^−5^ [61 × 10^−5^ to 208 × 10^−5^] at day 180) (Figures 1 and 3 and eTable 2 in Supplement 1). Levels were lower in SARS-CoV-2–naive recipients of the mRNA-1273 vaccine (37 × 10^−5^ [IQR, 23 × 10^−5^ to 71 × 10^−5^] at day 29; 54 × 10^−5^ [IQR, 31 × 10^−5^ to 90 × 10^−5^] at day 57; and 70 × 10^−5^ [IQR, 41 × 10^−5^ to 118 × 10^−5^] at day 180 [*P* < .001]). Levels of serum anti–spike IgA were better correlated with salivary-specific IgA among individuals with previous infection (remaining at day 180), rather than that of individuals without a prior infection (eFigure 1 in Supplement 1). In the latter groups, the correlation between blood and saliva IgA levels remained weak, with correlation coefficients substantially smaller than in individuals with previous infection (eFigure 1 in Supplement 1).

SARS-CoV-2 spike-specific saliva IgA levels remained more elevated 180 days after vaccination in individuals with previous infection compared with mRNA-1273–vaccinated individuals (104 × 10^−5^ [IQR, 61 × 10^−5^ to 208 × 10^−5^] vs 70 × 10^−5^ [IQR, 41 × 10^−5^ to 118 × 10^−5^]; *P* < .001) (Figure 1 and eTable 2 in Supplement 1). In individuals without prior infection, compared with day 1, increases of specific salivary IgA levels were substantially smaller, particularly after BNT162b2 vaccination, reaching statistical significance only at day 57 (36 × 10^−5^ [IQR, 19 × 10^−5^ to 79 × 10^−5^] vs 49 × 10^−5^ [IQR, 25 × 10^−5^ to 103 × 10^−5^]; *P* = .01) (Figure 3). In mRNA-1273–vaccinated individuals, specific saliva IgA levels increased significantly, albeit modestly, after the first (day 29 vs day 1, 1.4-fold [95% CI, 1.3- to 1.6-fold] change of geometric mean; *P* < .001) (eTable 3 in Supplement 1) and second vaccine doses (day 57 vs day 29, 1.3-fold [95% CI 1.1- to 1.5-fold] change of geometric mean; *P* < .001) (Figure 3 and eTable 3 in Supplement 1). In parallel, specific serum IgA levels increased in the same participants by 64.5-fold at day 29 vs day 1 (95% CI, 54.8- to 75.8-fold) and 182.6-fold at day 57 vs day 1 (95% CI, 154.3- to 216.1-fold) (eTable 1 in Supplement 1). Similar to IgG, salivary anti–spike IgA levels were significantly higher at day 180 in the mRNA-1273 group, compared with the BNT162b2 group (70 × 10^−5^ [IQR, 41 × 10^−5^ to 118 × 10^−5^] vs 43 × 10^−5^ [IQR, 24 × 10^−5^ to 84 × 10^−5^]; *P* < .001) (Figure 1). By definition, anti–nucleocapsid IgG levels were below the positivity threshold in individuals assigned to the mRNA-1273 group. Nevertheless, to verify whether asymptomatic infections might explain the late increase in anti–spike saliva IgA in the mRNA-1273 group, we split individuals in 2 groups according to anti–nucleocapsid IgG levels (SARS-CoV-2 nucleocapsid is not encoded by mRNA vaccines). As shown, anti–spike saliva IgA levels were not significantly elevated in individuals with anti–nucleocapsid serum IgG levels at day 180 above the 90th percentile of the distribution, compared with the others (eFigure 2 in Supplement 1). In conclusion, we did not find evidence that asymptomatic infections might explain the late increase in anti–spike saliva IgA in the mRNA-1273 group.

**Figure 4.**
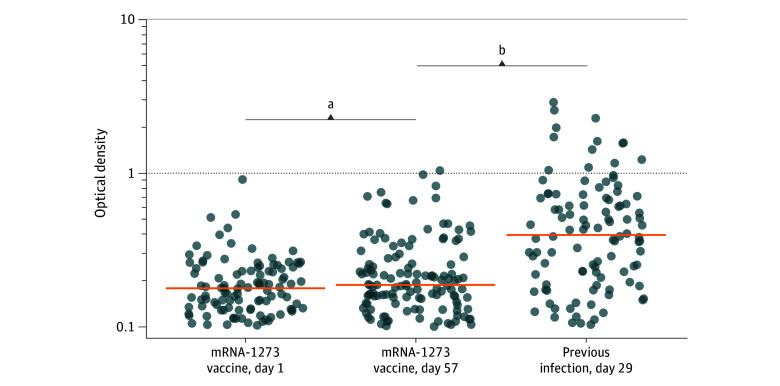
Multimeric Secretory Anti–Spike IgA After Vaccination in the Saliva of Individuals With Previous Infection Compared With SARS-CoV-2–Naive Individuals Measure of anti*–*spike secretory IgA by classic enzyme-linked immunosorbent assay, using antisecretory piece detection antibodies. SARS-CoV-2–naive individuals received the mRNA-1273 (Moderna) vaccine and previously infected individuals received the BNT162b2 (Pfizer-BioNTech) vaccine. Horizontal orange bars indicate median values. Results of the comparison between groups (Wilcoxon rank sum test) are shown. ^a^*P* ≤ .01, days 1 vs 57, for infection-naive participants receiving mRNA-1273 vaccine. ^b^*P* ≤ .001, day 57 for infection-naive participants receiving mRNA-1273 vaccine vs day 29 for previously infected individuals.

Finally, we verified whether bona fide mucosal secretory IgA could be locally induced at similar levels in SARS-CoV-2–naive individuals and those with previous infections by comparing SIgA levels at their peak of response in both groups (ie, days 57 and 29, respectively). As shown (Figure 4 and eTable 4 in Supplement 1), spike-specific SIgA levels were significantly more elevated in previously infected individuals (median optical density, 0.36 [IQR, 0.16-0.63] vs 0.16 [IQR, 0.10-0.22]; *P* < .001). Age-stratified Wilcoxon rank sum tests as sensitivity analyses for the comparisons between individuals with previous infection and SARS-CoV-2–naive individuals produced similar results as nonstratified tests, suggesting that differences between these groups were not confounded by age.

## Discussion

In this cohort study, we compared individuals with previous SARS-CoV-2 infection and SARS-CoV-2–naive individuals included in 2 large vaccination trials for their saliva and serum IgA and IgG antibody responses. Importantly, we limited the study to individuals vaccinated at the beginning of the pandemic to select a substantial number of confirmed SARS-CoV-2–naive participants to be included in the study.

Since SARS-CoV-2 infection became extremely prevalent in Europe and in the US, we speculate that some of the previous inconsistencies reported in the literature^3,4,5,6,7,8,9,10,11^ on saliva IgA responses might relate to difficulties recruiting participants who were truly SARS-CoV-2 naive. Many SARS-CoV-2 infections are indeed asymptomatic and even more so in the recent phases of virus circulation.^15^ Furthermore, infections do not always leave a lasting serologic signature.^15^ A recent study^11^ including a proportion of individuals with a history of SARS-CoV-2 infection concluded that mRNA vaccination induces by itself a robust mucosal antibody production. In this study, more robust responses were induced primarily in participants with previous infection (Figures 1 and 3).

However, a modest but significant increase in specific saliva IgA levels was observed in SARS-CoV-2–naive individuals receiving mRNA-1273 (Moderna) after the first and second administrations (Figure 1 and eTable 3 in Supplement 1). In parallel, specific serum IgA levels increased by 64.5-fold in the same participants (eTable 1 in Supplement 1). The correlations observed between serum and saliva IgA levels (eFigure 1 in Supplement 1) do not imply a blood origin for salivary IgA. Since the digital ELISA used in the study detects total anti–spike IgA, encompassing SIgA and possibly monomeric blood-borne IgA, we also measured electively secretory piece-bound SIgA to verify that bona fide SIgA levels were detected both in SARS-CoV-2–naive individuals and those with previous infection. However, specific SIgA levels were significantly more elevated in individuals with previous infection compared with SARS-CoV-2–naive individuals who received 2 antigenic stimulations (Figure 4).

Interestingly, we noted that anti–spike saliva IgA levels significantly increased between days 57 and 180 in the mRNA-1273 group (Figures 1 and 3), corresponding to an increase from 40 of 164 individuals (24%) to 55 of 159 (35%) with specific IgA levels above threshold (Figure 3). We reasoned that such a late variation could be related to asymptomatic SARS-CoV-2 infections. Data pertaining to anti–nucleocapsid saliva levels, by definition always below detection threshold in infection-naive individuals, were available to test that hypothesis. We found no correlation between anti–spike saliva IgA and serum anti–nucleocapsid IgG levels (eFigure 2 in Supplement 1) that could have pointed in the direction of asymptomatic infections to explain a late increase in anti–spike saliva IgA levels in the mRNA-1273 vaccine group. It appears unlikely as well that late seronegative SARS-CoV-2 infections would have only occurred in the same latter group. Finally, our observation that specific serum and saliva IgA levels were significantly higher in SARS-CoV-2–naive individuals at day 180 after vaccination with mRNA-1273 (Moderna) compared with BNT162b2 (Pfizer-BioNTech) (Figure 1) coincides with reports of more elevated antibody responses, which are associated with slightly lower hospitalization rates in the former group.^16,17^

### Limitations

This study has some limitations. We cannot determine whether the discrete increase in specific SIgA observed in some SARS-CoV-2–naive vaccinations is the result of asymptomatic or seronegative exposures to respiratory virus, mucosal seeding by rare antibody-secreting cells trafficking from vaccine-draining lymph nodes, or both. In addition, we did not include data on neutralizing antibodies; however, prior studies^2,18^ suggest that specific antibody levels are strongly correlated. The number of vaccine breakthrough cases reported in our cohort was too limited to address the association between SIgA levels and prevention of infection or transmission of SARS-CoV-2.

## Conclusion

The findings of this cohort study suggest that mRNA vaccination was associated with mucosal immunity in individuals with prior SARS-CoV-2 infection. Further studies are needed to determine the association between SIgA levels and prevention of infection or transmission.

## Notes

Supplement 1. **eTable 1.** Serum SARS-CoV-2 Spike-Specific IgG and IgA **eTable 2.** Salivary SARS-CoV-2 Spike-Specific IgG and IgA **eTable 3.** Intragroup Longitudinal Variations of Salivary SARS-CoV-2 Spike-Specific IgA **eTable 4.** SARS-CoV-2 Spike-Specific Secretory IgA (OD) **eTable 5.** *P* Values Corresponding to Data Analyses in Figures 2, 3, and 4 **eFigure 1.** Correlations Between Serum and Saliva IgG and IgA **eFigure 2.** No Serological Evidence for Asymptomatic Infection in Moderna-Vaccinated Individuals With Late Increase in Specific Saliva IgA **eMethods.** Digital ELISA Assay for SARS-CoV-2 Spike-Specific IgA

Supplement 2.
Data Sharing Statement
