# Supplementary material for: Serum and Salivary IgG and IgA Response After COVID-19 Messenger RNA Vaccination
Source: JAMA Netw Open. 2024 Apr 23;7(4):e248051. doi: 10.1001/jamanetworkopen.2024.8051 (PMC11040412; doi:10.1001/jamanetworkopen.2024.8051)
Supplement: Supplement 1. — eTable 1. Serum SARS-CoV-2 Spike-Specific IgG and IgA eTable 2. Salivary SARS-CoV-2 Spike-Specific IgG and IgA eTable 3. Intragroup Longitudinal Variations of Salivary SARS-CoV-2 Spike-Specific IgA eTable 4. SARS-CoV-2 Spike-Specific Secretory IgA (OD) eTable 5. P Values Corresponding to Data Analyses in Figures 2, 3, and 4 eFigure 1. Correlations Between Serum and Saliva IgG and IgA eFigure 2. No Serological Evidence for Asymptomatic Infection in Moderna-Vaccinated Individuals With Late Increase in Specific Saliva IgA eMethods. Digital ELISA Assay for SARS-CoV-2 Spike-Specific IgA [file jamanetwopen-e248051-s001.pdf]

Supplementary Online Content

Gorochov G, Ropers J, Launay O, et al. Serum and salivary IgG and IgA response after COVID-19 messenger RNA vaccination. *JAMA Netw Open*. 2024;7(4):e248051. doi:10.1001/jamanetworkopen.2024.8051

eTable 1. Serum SARS-CoV-2 Spike-Specific IgG and IgA
eTable 2. Salivary SARS-CoV-2 Spike-Specific IgG and IgA
eTable 3. Intragroup Longitudinal Variations of Salivary SARS-CoV-2 Spike-Specific IgA
eTable 4. SARS-CoV-2 Spike-Specific Secretory IgA (OD)
eTable 5*.* *P* Values Corresponding to Data Analyses in Figures 2, 3, and 4
eFigure 1. Correlations Between Serum and Saliva IgG and IgA
eFigure 2. No Serological Evidence for Asymptomatic Infection in Moderna-Vaccinated Individuals With Late Increase in Specific Saliva IgA
eMethods. Digital ELISA Assay for SARS-CoV-2 Spike-Specific IgA

This supplementary material has been provided by the authors to give readers additional information about their work.

© 2024 Gorochov G et al. *JAMA Network Open*.

eTable 1. Serum SARS-CoV-2 Spike-Specific IgG and IgA

Abbreviation: IQR: interquartile range

	D1	D29	D57	D180
**mRNA-1273, N = 172**				
IgG, Median (IQR)	0.3 (0.2 – 0.7)	285.4 (152.4 – 493.6)	2,090.9 (1,250.6 – 3,051.4)	395.4 (201.4 – 621.3)
Missing	0	0	0	0
IgA, Median (IQR)	2.9 (1.9 – 5.5)	217.1 (111.5 – 474.1)	603.9 (315.3 – 1,209.1)	119.2 (53.5 – 260.0)
Missing	0	0	0	0
**BNT162b2, N = 135**				
IgG, Median (IQR)	0.3 (0.2 – 0.6)	117.5 (57.4 – 243.0)	1,292.4 (794.2 – 2,031.6)	247.9 (144.5 – 380.0)
Missing	0	0	0	1
IgA, Median (IQR)	3.4 (2.0 – 6.7)	74.4 (40.0 – 164.0)	249.4 (144.3 – 483.3)	63.0 (33.0 – 151.8)
Missing	1	0	0	1
**Previously Infected, N = 120**				
IgG, Median (IQR)	132.7 (38.8 – 276.2)	6,888.8 (4,000.2 – 12,696.6)	4,859.1 (2,850.4 – 7,970.2)	2,110.9 (698.8 – 4,493.4)
Missing	0	0	4	5
IgA, Median (IQR)	103.2 (44.8 – 215.0)	2,211.5 (858.9 – 4,497.9)	1,438.6 (705.1 – 2,753.1)	813.4 (409.1 – 1,880.7)
Missing	0	0	4	5

Quantitative results expressed in standardized binding antibody units per mL (BAU/mL).

© 2024 Gorochov G et al. *JAMA Network Open*.

Table 2. Salivary SARS-CoV-2 Spike-Specific IgG and IgA

	D1	D29	D57	D180
**mRNA-1273, N = 172**				
IgG saliva, normalized*, Median (IQR)	0.00002 (0.00001 – 0.00007)	0.00060 (0.00030 – 0.00109)	0.00440 (0.00278 – 0.00725)	0.00081 (0.00040 – 0.00137)
Missing	5	1	3	13
IgA-saliva, normalized**,Median (IQR)	0.00026 (0.00015 – 0.00048)	0.00037 (0.00023 – 0.00071)	0.00054 (0.00031 – 0.00090)	0.00070 (0.00041 – 0.00118)
Missing	5	2	8	13
**BNT162b2, N = 135**				
IgG saliva, normalized*, Median (IQR)	0.00003 (0.00001 – 0.00009)	0.00028 (0.00011 – 0.00061)	0.00292 (0.00140 – 0.00544)	0.00045 (0.00025 – 0.00073)
Missing	0	0	1	2
IgA saliva, normalized**, Median (IQR)	0.00036 (0.00019 – 0.00079)	0.00044 (0.00021 – 0.00070)	0.00049 (0.00025 – 0.00103)	0.00043 (0.00024 – 0.00084)
Missing	6	8	14	2
**Previously Infected, N = 120**				
IgG saliva, normalized*, Median (IQR)	0.00021 (0.00009 – 0.00059)	0.00990 (0.00340 – 0.01915)	0.00935 (0.00387 – 0.01900)	0.00382 (0.00152 – 0.01085)
Missing	3	2	10	4
IgA saliva, normalized**, Median (IQR)	0.00052 (0.00030 – 0.00105)	0.00155 (0.00069 – 0.00387)	0.00107 (0.00063 – 0.00217)	0.00104 (0.00061 – 0.00208)
Missing	7	6	20	4

Abbreviation: IQR: interquartile range

*Normalized IgG = ng (specific IgG)/ng (total IgG)

**Normalized IgA = AU (specific IgA)/ng (total IgA)

© 2024 Gorochov G et al. *JAMA Network Open*.

eTable 3. Intragroup Longitudinal Variations of Salivary SARS-CoV-2 Spike-Specific IgA

		Missing	p-value[1]	Fold change (CI 95%)
**mRNA-1273, N = 172**				
D1	D29	6	<0.001	1.41 (1.25 - 1.59)
D1	D57	12	<0.001	1.89 (1.64 - 2.17)
D1	D180	18	<0.001	2.45 (2.09 - 2.87)
D29	D57	9	<0.001	1.30 (1.14 - 1.48)
D57	D180	21	0.002	1.27 (1.08 - 1.50)
**BNT162b2, N = 135**				
D1	D29	10	0.25	1.11 (0.94 - 1.31)
D1	D57	17	0.01	1.29 (1.08 - 1.54)
D1	D180	8	0.41	1.15 (0.93 - 1.42)
D29	D57	15	0.07	1.17 (0.96 - 1.42)
D57	D180	15	0.16	0.86 (0.68 - 1.09)
**Previously Infected, N = 120**				
D1	D29	10	<0.001	2.86 (2.31 - 3.54)
D1	D57	23	<0.001	1.89 (1.52 - 2.36)
D1	D180	11	<0.001	1.80 (1.45 - 2.25)
D29	D57	21	<0.001	0.64 (0.53 - 0.79)
D57	D180	21	0.28	0.93 (0.76 - 1.15)

Abbreviation: CI: confidence interval

[1]Wilcoxon rank sum test for paired data

© 2024 Gorochov G et al. *JAMA Network Open*.

eTable 4. SARS-CoV-2 Spike-Specific Secretory IgA (OD)

	**mRNA-1273 D57**, N = 172	**Previously infected D29**, N = 120	**p-value**[1]
OD, Median (IQR)	0.16 (0.10 – 0.22)	0.36 (0.16 – 0.63)	<0.001
Missing	2	2	

Abbreviation: IQR: interquartile range; OD: optical density

[1]Wilcoxon rank sum test; [2]Wilcoxon rank sum test stratified on age

© 2024 Gorochov G et al. *JAMA Network Open*.

eTable 5. *P* Values Corresponding to Data Analyses in Figures 2, 3, and 4

	Missing	Median (IQR)	D1	D29	D57	D180
**mRNA-1273, N = 172**						
IgG saliva, normalized*						
D1	5	0.00002 (0.00001 - 0.00007)	-	<0.001	<0.001	<0.001
D29	1	0.0006 (0.0003 - 0.00109)	-	-	<0.001	0.004
D57	3	0.0044 (0.00278 - 0.00725)	-	-	-	<0.001
D180	13	0.00081 (0.0004 - 0.00137)	-	-	-	-
IgA saliva, normalized**						
D1	5	0.00026 (0.00015 - 0.00048)	-	<0.001	<0.001	<0.001
D29	2	0.00037 (0.00023 - 0.00071)	-	-	<0.001	0.004
D57	8	0.00054 (0.00031 - 0.0009)	-	-	-	<0.001
D180	13	0.0007 (0.00041 - 0.00118)	-	-	-	-
**BNT162b2, N = 135**						
IgG saliva, normalized*						
D1	0	0.00003 (0.00001 - 0.00009)	-	<0.001	<0.001	<0.001
D29	0	0.00028 (0.00011 - 0.00061)	-	-	<0.001	<0.001
D57	1	0.00292 (0.0014 - 0.00544)	-	-	-	<0.001
D180	2	0.00045 (0.00025 - 0.00073)	-	-	-	-
IgA saliva, normalized**						
D1	6	0.00036 (0.00019 - 0.00079)	-	0.25	0.01	0.41
D29	8	0.00044 (0.00021 - 0.0007)	-	-	0.07	0.64
D57	14	0.00049 (0.00025 - 0.00103)	-	-	-	0.16
D180	2	0.00043 (0.00024 - 0.00084)	-	-	-	-
**Previously Infected, N = 120**						
IgG saliva, normalized*						
D1	3	0.00021 (0.00009 - 0.00059)	-	<0.001	<0.001	<0.001
D29	2	0.0099 (0.0034 - 0.01915)	-	-	0.26	<0.001

© 2024 Gorochov G et al. *JAMA Network Open*.

	Missing	Median (IQR)	D1	D29	D57	D180
D57	10	0.00935 (0.00387 - 0.019)	-	-	-	<0.001
D180	4	0.00382 (0.00152 - 0.01085)	-	-	-	-
IgA saliva, normalized**						
D1	7	0.00052 (0.0003 - 0.00105)	-	<0.001	<0.001	<0.001
D29	6	0.00155 (0.00069 - 0.00387)	-	-	<0.001	<0.001
D57	20	0.00107 (0.00063 - 0.00217)	-	-	-	0.28
D180	4	0.00104 (0.00061 - 0.00208)	-	-	-	-

mRNA-1273, N = 172

D180 IgA saliva, normalized**	IgG N < 90th percentile (Missing = 12)	IgG N > 90th percentile (Missing = 1)	p
Median (IQR)	0.00067 (0.00039 - 0.00111)	0.00085 (0.00057 - 0.00143)	0.10

Abbreviation: IQR: interquartile range
*Normalized IgG = ng (specific IgG)/ng (total IgG)
**Normalized IgA = AU (specific IgA)/ng (total IgA)

© 2024 Gorochov G et al. *JAMA Network Open*.

eFigure 1. Correlations Between Serum and Saliva IgG and IgA

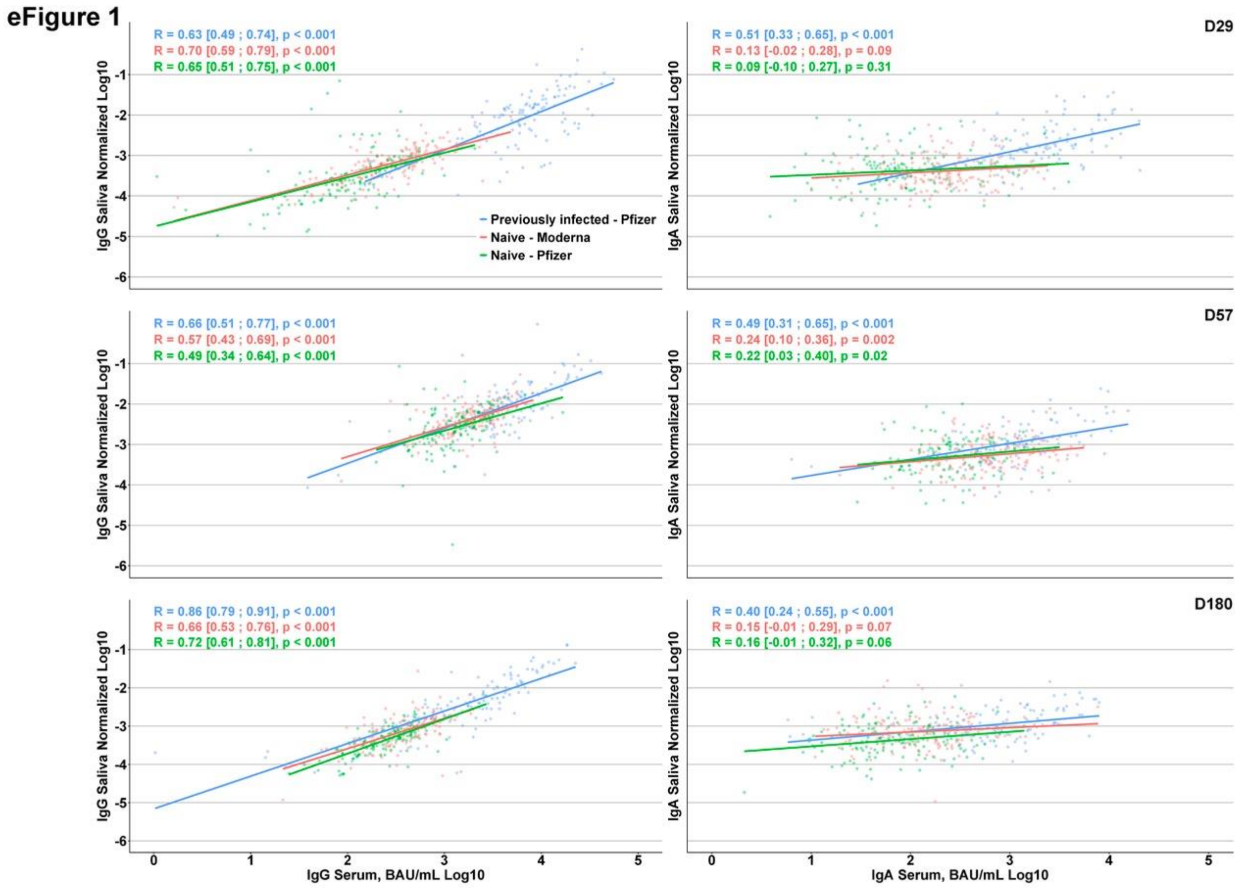

Correlations between serum and saliva IgG (left column), serum and saliva IgA (right) at indicated times (day 29, 57 and 180) after vaccination in BNT162b2-vaccinated previously infected individuals (PI, blue), mRNA-1273-vaccinated naïve individuals (M, red) and BNT162b2-vaccinated naïve individuals (P, green). Regression lines are standard linear regressions. Spearman rank correlations are shown on top of each graph along with 95% confidence intervals and p-values.

© 2024 Gorochov G et al. *JAMA Network Open*.

eFigure 2. No Serological Evidence for Asymptomatic Infection in Moderna-Vaccinated Individuals With Late Increase in Specific Saliva IgA

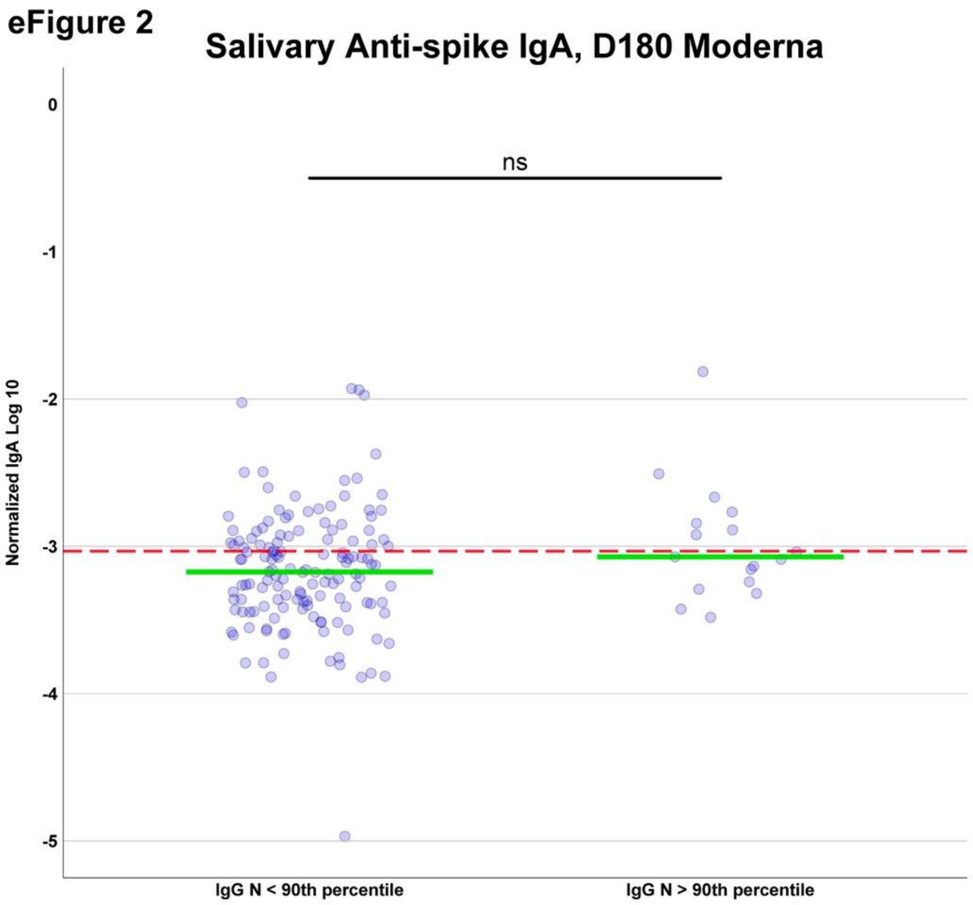

By definition all individuals tested here had antinucleocapsid antibodies below detection threshold. Levels of salivary anti-spike IgA at day 180 (measured by digital Elisa) among SARS-CoV-2-naive individuals following 2 mRNA-1273 injections split in two groups according to antinucleocapsid signals recorded. Groups were formed according to serum Elisa signals against nucleocapside measured by classical Elisa at day 180 in naïve individuals (being lower or greater than the 90th percentile of the anti-N distribution). Dotted line indicates threshold value for specific IgA positivity computed arbitrarily as the mean + 1 standard deviation of measurements performed at baseline in naive individuals. Green plain lines indicate median values of antinucleocapsid signals. Results of comparison between groups (Wilcoxon rank sum test) are shown with $p > 0.05$: ns.

eMethods. Digital ELISA Assay for SARS-CoV-2 Spike-Specific IgA

© 2024 Gorochov G et al. *JAMA Network Open*.

In the first step of the assay, 25 µL of SARS-CoV-2 spike-coupled beads (Bead Reagent from the SARS-CoV-2 spike IgG Advantage Assay kit) were incubated with 100 µL of diluted saliva in a 96-well conical bottom plate for 30 min at 30°C at 800 rpm. The plate was washed on a Simoa microplate washer equipped with a magnet, and the beads were incubated with 100 µL of detector reagent for 10 minutes at 30°C at 800 rpm. After a new washing step, beads were incubated with the SBG reagent and incubated at 30°C at 800 rpm for 10 minutes. The beads were then washed twice in buffer B at 800 rpm for 1 minute. Buffer was then removed and the beads were dried for 10 minutes on the magnet. The plate was then transferred to the SR-X instrument with RGP reagent and read according to a home-brew analysis protocol. The concentration of each sample was calculated based on the four-parameter logistic fitting model generated with the calibrators. Signals are expressed in binding antibody units (BAU)/mL.
Limit of Detection: Lower Limit of Detection (LOD) is calculated as 2.5 Standard Deviations above the background (mean of calibrator blanks).
Mean LOD: 0.074 BAU/mL (Range: 0.030 – 0.134 BAU/mL)
Limit of Quantification: Lower Limit of Quantification (LLOQ) is the highest concentration of calibrator with ≤ 20% pooled CV.
Mean LLOQ: 0.093 (Range: 0.067 – 0.148 BAU/mL)
Functional LLOQ is the Analytical LLOQ multiplied by the Minimum Required Dilution (1:100): 9.25 BAU/mL
Data normalization: To minimize the impact of variations between runs, all sample concentrations were normalized. Six saliva samples diluted 1:100 were used as controls in each run performed on the SR-X analyser (Quanterix®). The average concentration of controls and the corresponding AEB values (AEB: average enzymes per bead is the unit of measurement for Simoa) were used to construct a new calibration curve for each analysis. The mean concentration corrected for MRD (BAU/mL) and the coefficient of variation (% CV) based on concentration are reported in the table below.

**Experiments**	**Control** 1	**Control** 2	**Control** 3	**Control** 4	**Control** 5	**Control** 6
Mean (BAU/mL)	16	95	146	241	1248	1976
Range	13 - 20	76 - 123	121 - 163	203 - 280	893 - 1665	1398 - 2843
CV	14%	17%	11%	12%	18%	22%

Anti-SARS-CoV-2 spike IgA binding capacity in samples is related to antibody concentration but also to affinity and avidity (dimer *vs* monomer) effects. IgA are mostly dimeric in saliva. For that reason, we chose here to express anti-Spike IgA data as BAU (binding antibody units) and not in concentration (ng/ml). Although useful, available monomeric recombinant anti-Spike IgA antibodies cannot be used as calibrators to measure accurate concentrations. Furthermore, after vaccination or infection, the anti-Spike IgA response is polyclonal and the overall binding capacity of this heterogeneous population of antibodies may not be comparable to that of a monoclonal IgA used as a reference.

© 2024 Gorochov G et al. *JAMA Network Open*.
