# Supplementary material for: Serum and Salivary IgG and IgA Response After COVID-19 Messenger RNA Vaccination
Source: JAMA Netw Open. 2024 Apr 23;7(4):e248051. doi: 10.1001/jamanetworkopen.2024.8051 (PMC11040412; doi:10.1001/jamanetworkopen.2024.8051)
Supplement: Supplement 2. — Data Sharing Statement [file jamanetwopen-e248051-s002.pdf]

Data Sharing Statement

Gorochov. Serum and Salivary IgG and IgA Response After COVID-19 Messenger RNA Vaccination. *JAMA Netw Open*. Published April 23, 2024.
doi:10.1001/jamanetworkopen.2024.8051

Data
Data available: No

Additional Information
Explanation for why data not available: The procedures carried out with the French data privacy authority (CNIL, Commission nationale de l'informatique et des libertés) do not provide for the transmission of the database, nor do the information and consent documents signed by the patients. Consultation, by the editorial board or interested researchers, of individual participant data that underlie the results reported in this article may nevertheless be considered after de-identification, subject to prior determination of the terms and conditions of such consulta-tion and in respect for compliance with the applicable regulations.
